# Effect of introduction of a standardised peri-operative protocol on CSF shunt infection rate: a single-centre cohort study of 809 procedures

**DOI:** 10.1007/s00381-018-3953-0

**Published:** 2018-08-21

**Authors:** Osama Omrani, Jody O’Connor, John Hartley, Greg James

**Affiliations:** 1 Department of Neurosurgery, Great Ormond Street Hospital, Great Ormond Street, London, WC1N 3JH UK; 2 Barts and the London School of Medicine and Dentistry, London, UK; 3 Department of Microbiology, Great Ormond Street Hospital, Great Ormond Street, London, WC1N 3JH UK; 4 Great Ormond Street Institute of Child Health, University College London, London, UK

**Keywords:** Hydrocephalus, Ventriculoperitoneal shunt, Intracranial infection, Paediatrics

## Abstract

**Purpose:**

Shunt infection is a major problem in paediatric neurosurgery. Our institution introduced a mandatory shunt protocol with the aim of reducing infection rate.

**Methods:**

A retrospective cohort study including consecutive patients undergoing permanent shunt operations (primary insertion and revision) across two study periods: 3 years immediately prior (2009–2012) and 3 years immediately after (2012–2015) protocol introduction*.* Absolute and relative risk reductions (ARR/RRR) and Chi-square statistical analysis was used alongside logistic regression, where any single factor with *p* ≤ 0.20 included in the multivariate model, producing an odds ratio (OR).

**Results:**

Eight hundred nine operations in 504 children were identified (442 pre-protocol, 367 post). Overall infection rate decreased from 5.43% (24/442) pre-protocol to 3.27% (12/367) post-protocol (ARR = 2.16%, RRR = 39.8%, NNT = 46.3, *p* = 0.138), which did not reach statistical significance. For primary shunt insertions, infection rate reduced from 3.63 to 2.55% (ARR = 1.08%, RRR = 29.8%, NNT = 92.6, *p* = 0.565), whilst for revisions, it reduced from 6.83 to 3.81% (ARR = 3.02%, RRR 44.2%, NNT = 33.1, *p* = 0.156). Multivariate logistic regression showed that surgeon experience was a statistically significant predictor of infection, whilst responsible pathogens and latency were similar across the pre- and post-protocol groups.

**Conclusion:**

The protocol reduced overall infection rate in primary and revision shunt operations and we recommend paediatric units consider introducing a similar protocol for these procedures.

## Introduction

Implanted CSF shunts are used extensively in the management of hydrocephalus, and shunt-associated infection is a major source of morbidity in paediatric neurosurgical practice [1]. Single-centre shunt infection rates range between 6 and 15% [2, 3]. A recent multi-centre study, which included 1036 patients from 6 centres in the USA, reported an overall shunt surgery infection rate of 11% [4].

Shunt infection is associated with considerable mortality and morbidity. Infection-related mortality has been reported to range from 1.5 to 22% [2], with those surviving facing the risk of significant morbidities such as permanent neurological deficit, cognitive impairment, and epilepsy [5]. Shunt infections are treated with intravenous and intra-ventricular antibiotics, which come with associated side effects, lengthy hospital stays, and the need for additional surgical procedures [6].

These serious implications, coupled with the fact that shunt operations are the most common paediatric neurosurgical procedure performed, have made targeting and minimising shunt infections a key concern. Recent literature produced by the Hydrocephalus Clinical Research Network has suggested that the development and implementation of a standardised, stepwise protocol governing paediatric shunt procedures can have a significant effect on shunt infection rates [7, 8].

Motivated by this important work, we developed a comprehensive and mandatory “shunt protocol” for our institution, with guidance on pre-, intra-, and post-operative surgical and nursing management, with the aim of reducing shunt surgery-associated infections. We report our experience and early results following introduction of this protocol, by comparing outcomes following introduction to the cohort of patients treated immediately prior.

## Methods

### Study design

Using our institution’s computerised prospective neurosurgical database that maintains details of patients’ demographics, operative details, and complications, we identified all patients who underwent a permanent ventriculoperitoneal, ventriculoatrial, or ventriculopleural shunt procedure within our study period. The protocol was introduced on the 1st of July 2012, which was used as the cut-off point to produce two cohorts containing consecutive cases, one cohort including all relevant shunt procedures 3 years immediately prior to this date (1st July 2009–31st June 2012) and the other cohort including those 3 years immediately after (1st July 2012–31st June 2015). Procedures were also subdivided into primary insertion and shunt revisions. “Primary insertion” was defined as insertion of a shunt into a child with no pre-existing CSF diversion hardware—either externalised drains or shunt. VP shunt insertions in children who had undergone previous non-CSF shunt surgeries, such as endoscopic third ventriculostomies or CNS tumour resections, were included in this category. “Revision” was defined as any surgery on a child with a pre-existing, internal CSF shunt whereby one or more (including all) of the components of the shunt were changed, but that the child continued to have a permanent, internalised shunt at the end of the procedure (i.e. shunt removals and externalisations were not included in the analysis). We excluded patients having “internalisation” procedures (i.e. insertion of shunt hardware at the same operation of removal of external drainage systems).

Infections were identified using the complications contemporaneously recorded on the neurosurgical database, and confirmed by interrogation of case notes and our separate pathology database. The minimum follow-up was 12 months post-surgery. The following criteria were used to define CSF shunt infection: CSF microscopy or culture that yielded an organism or CSF pleocytosis associated with fever, shunt malfunction, or neurological symptoms that required shunt removal and subsequent antimicrobial treatment. Superficial incisional or deep incisional infections that did not require shunt removal were not classed as CSF shunt infection.

### Shunt protocol

A multidisciplinary team in our institution, including consultant neurosurgeons, microbiologists, and specialist nurses produced and reviewed the stepwise protocol using a combination of relevant literature and collective clinical experience [7, 9–12]. The protocol identified factors relevant to the four phases of each procedure, i.e. pre-operative, intra-operative, post-operative, and pre-discharge factors.

**Fig. 1:**
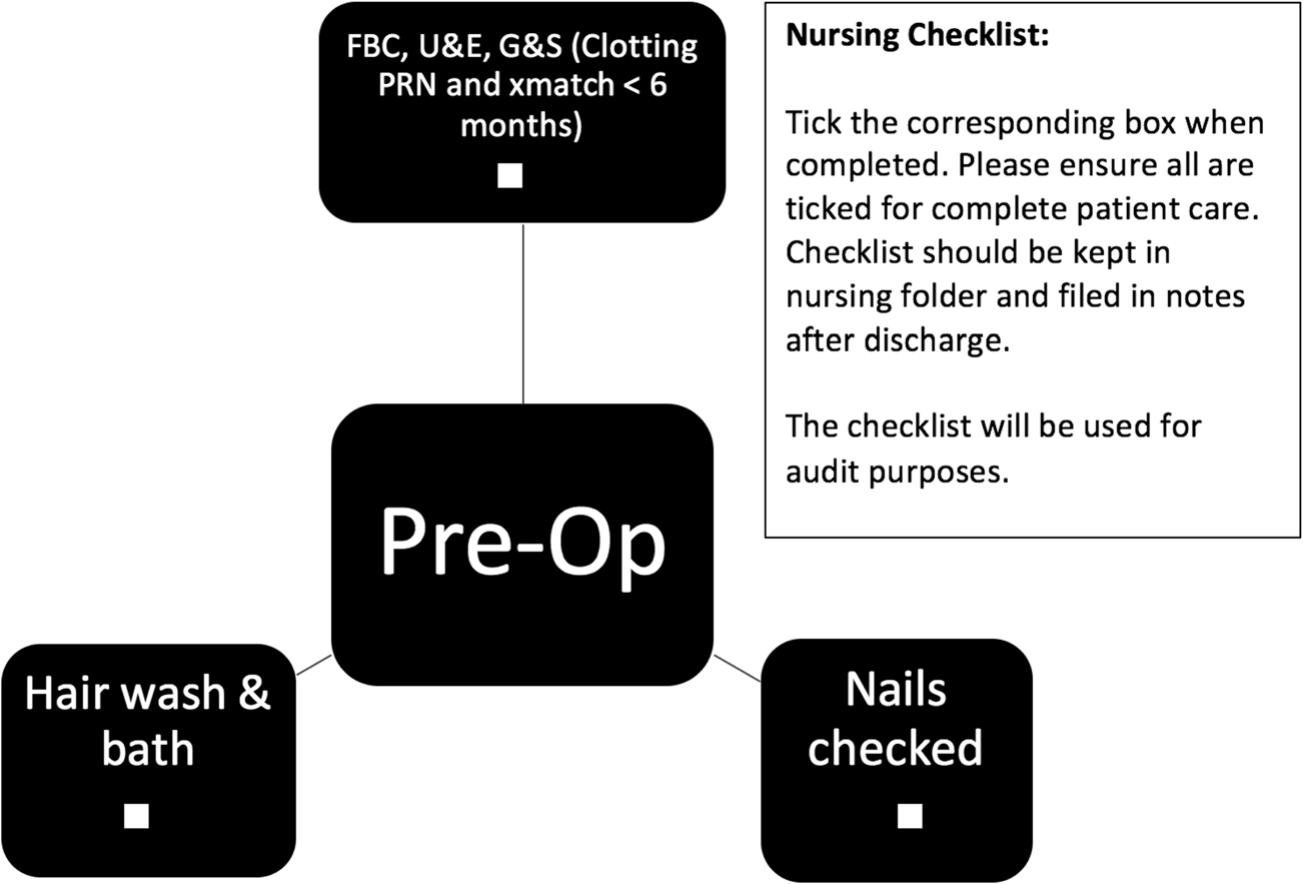
Pre-operative protocol check list

**Fig. 2:**
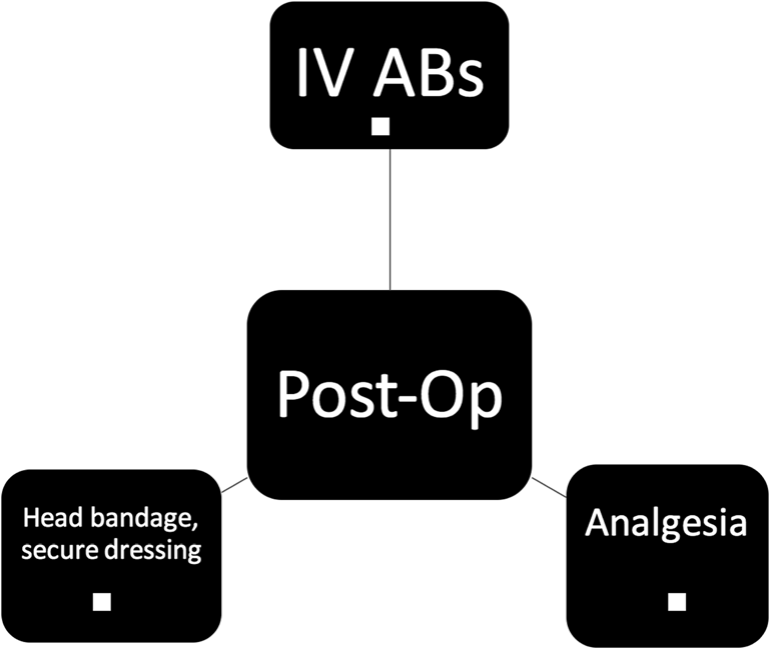
Post-operative protocol check list

**Fig. 3:**
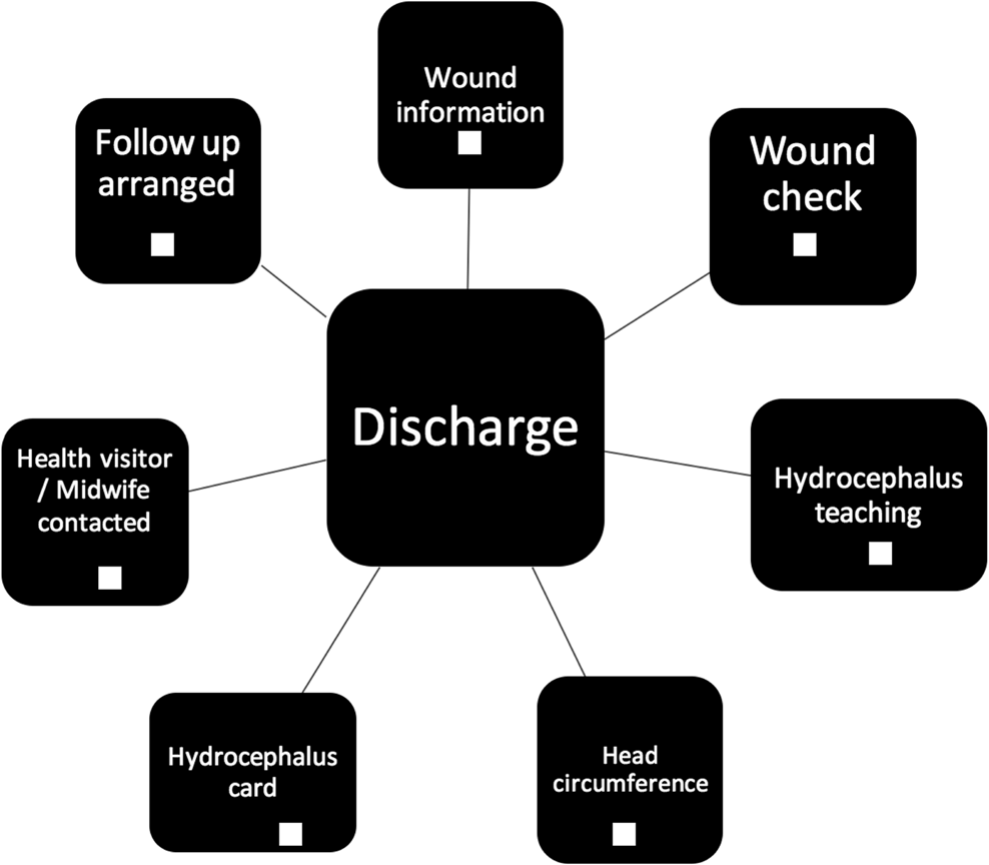
Discharge protocol check list

**Fig. 4:**
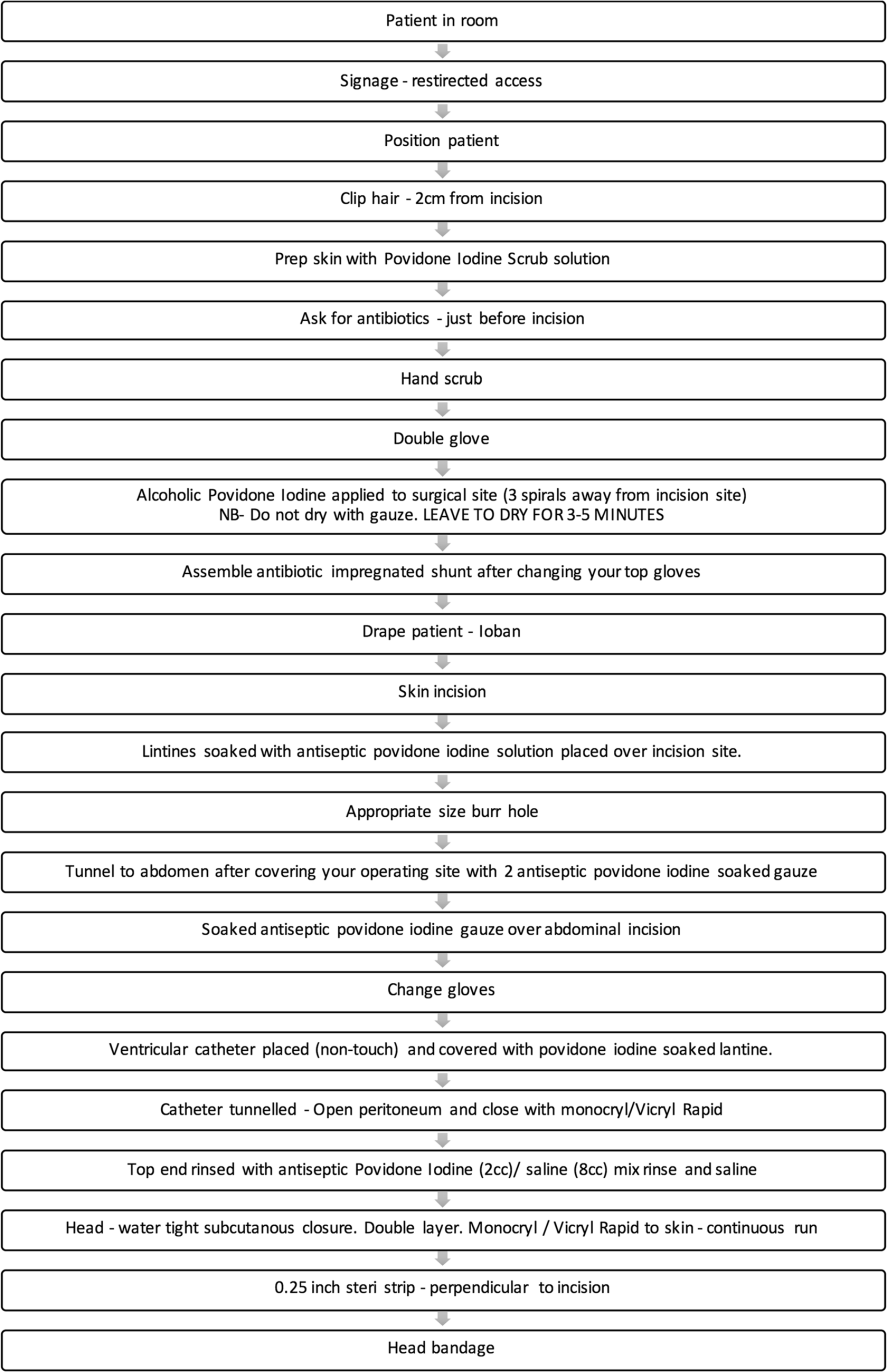
Intra-operative protocol check list

Pre-operative care factors designed to reduce infection rate include a chlorhexidine bath and hair wash, ensuring patients have short, clean nails with no nail varnish and are dressed in appropriate theatre gowns with cotton underwear (Fig. 1). Intra-operative care included guidelines on number of persons in theatre, hair clipping, site preparation, draping, intra-operative technique, wound closure and dressings, as well as intra-operative antibiotics and patient warming (Fig. 2). Post-operative and pre-discharge factors focused on washing, wound care, and antibiotic administration (Figs. 3 and 4).

Compliance at all four stages was monitored with checklists provided to ward and operating room nursing staff. All children admitted for shunt surgery have these checklists automatically added to their clinical notes.

### Statistical analysis

Pearson chi-square test and two-tailed *T* test were used to compare study group characteristics and infection rate before and after the introduction of the protocol, with further subgroup analysis comparing the effect of protocol introduction on primary and revision procedures. Univariate binary logistic regression was used to investigate for an association between infection and the following factors: age at procedure, gender of patient, type of procedure, use of protocol, grade of operating surgeon, nature of operation (emergency or routine), and presenting diagnosis. Any factor with at least a weak association (*p* ≤ 0.2) was then included in the multivariate logistic regression model. The effect of the protocol and age at procedure were then forced into the model. Age was included due to the established association between young age and shunt infection in literature [1, 13, 14]. Latency between date of procedure and infection diagnosis and cultured organism data was recorded in order to identify the most common pathogens and to note any variation in frequency or latency between the pre and post-protocol groups. Statistical analysis was carried using Microsoft Excel (Microsoft, Inc.) and SPSS statistical software (SPSS, Inc.).

## Results

### Study group characteristics

Eight hundred nine procedures performed in 504 patients matched our inclusion criteria, with 442 procedures in the pre-protocol cohort and 367 in the post-protocol cohort. Population demographics are tabulated in Table 1. There were no statistically significant differences noted between the two cohorts in terms of age at procedure, gender, type of procedure, or grade of operating surgeon.

**Table 1:** Demographics. *IVH* intra-ventricular haemorrhage, *IIH* idiopathic intracranial hypertension, *SD* standard deviation

	Overall	Pre-protocol	Post-protocol
Number of procedures	809	442	367
Mean age in months ± SD	55.8 ± 62.8	59.4 ± 63.7	49.8 ± 60.4
Gender			
Male	435 (53.8%)	225 (50.9%)	210 (57.2%)
Female	374 (46.2%)	217 (49.1%)	157 (42.8%)
Procedure type			
Primary shunt insertion	350 (43.3%)	193 (43.7%)	157 (42.8%)
Shunt revision	459 (56.7%)	249 (56.3%)	210 (57.2%)
Diagnosis			
Tumour	123 (15.2%)	72 (16.3%)	65 (13.9%)
Post-IVH	190 (23.5%)	84 (19.0%)	106 (28.9%)
Spina bifida	78 (9.6%)	38 (8.6%)	40 (10.9%)
Craniofacial or craniocervical	57 (7%)	35 (7.9%)	22 (6.0%)
IIH	38 (4.7%)	26 (5.9%)	12 (3.3%)
Post-infection	38 (4.7%)	20 (4.5%)	18 (4.9%)
Aqueduct stenosis	27 (3.3%)	16 (3.6%)	11 (3.0%)
Vascular disorder	13 (1.6%)	8 (1.8%)	5 (1.4%)
Trauma	8 (1.0%)	6 (1.4%)	2 (0.5%)
Epilepsy	1 (0.1%)	1 (0.2%)	0 (0.0%)
Other	236 (29.2%)	136 (30.8%)	100 (27.2%)
Grade of operating surgeon			
Consultant	496 (61.3%)	271 (61.3%)	225 (61.3%)
Resident/fellow	313 (38.7%)	171 (38.7%)	142 (38.7%)

**Fig. 5:**
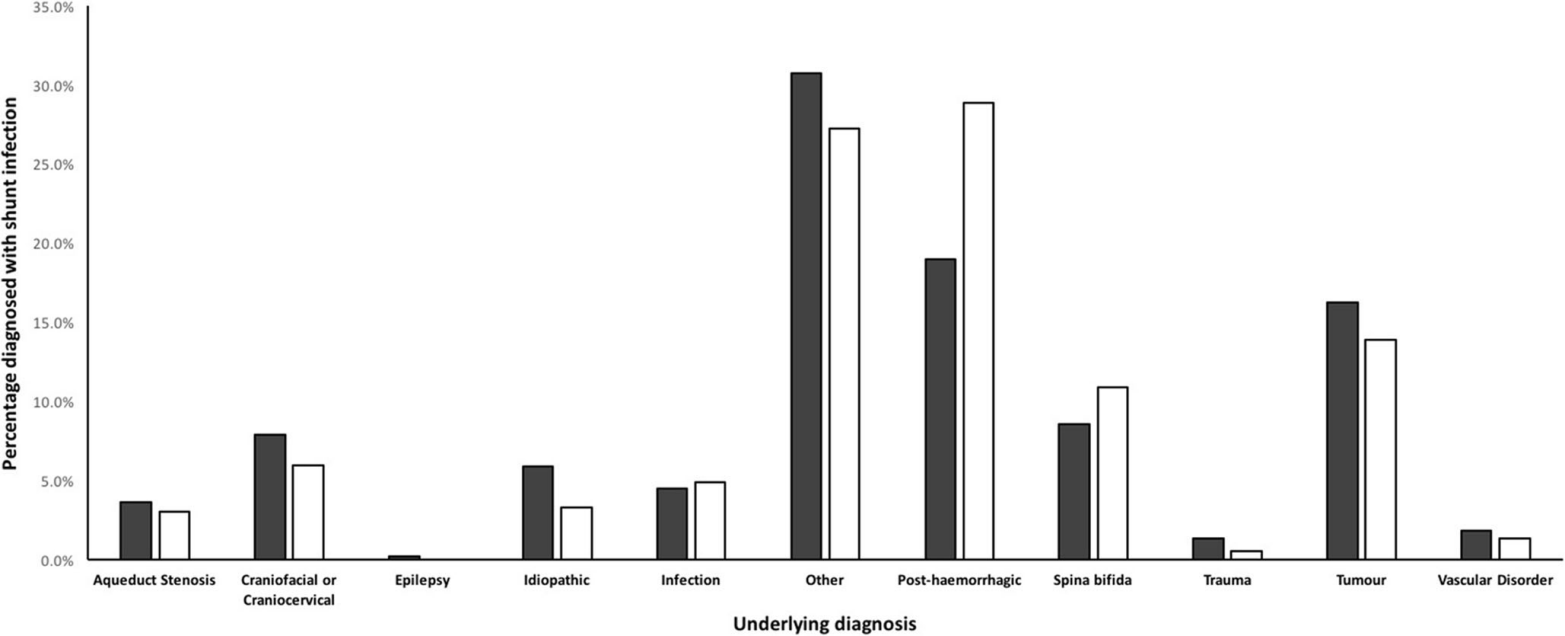
Breakdown of hydrocephalus aetiology in both cohorts

Generally, there was good equivalence between the two cohorts in terms of aetiology, with no statistically significant differences noted. The most frequently identified aetiology was post-haemorrhagic hydrocephalus, representing 23.5% of all patients, followed by tumour-induced hydrocephalus making up 15.2% of patients (Fig. 5).

### Shunt infection rate

A total of 36 shunt infections in 809 procedures were identified across the study period, i.e. 4.45% of all shunt procedures identified. In the primary shunt insertion subgroup, there were 11/350 infections (3.14%), whilst in the shunt revision subgroup, there were 25/459 infections (5.45%).

**Fig. 6:**
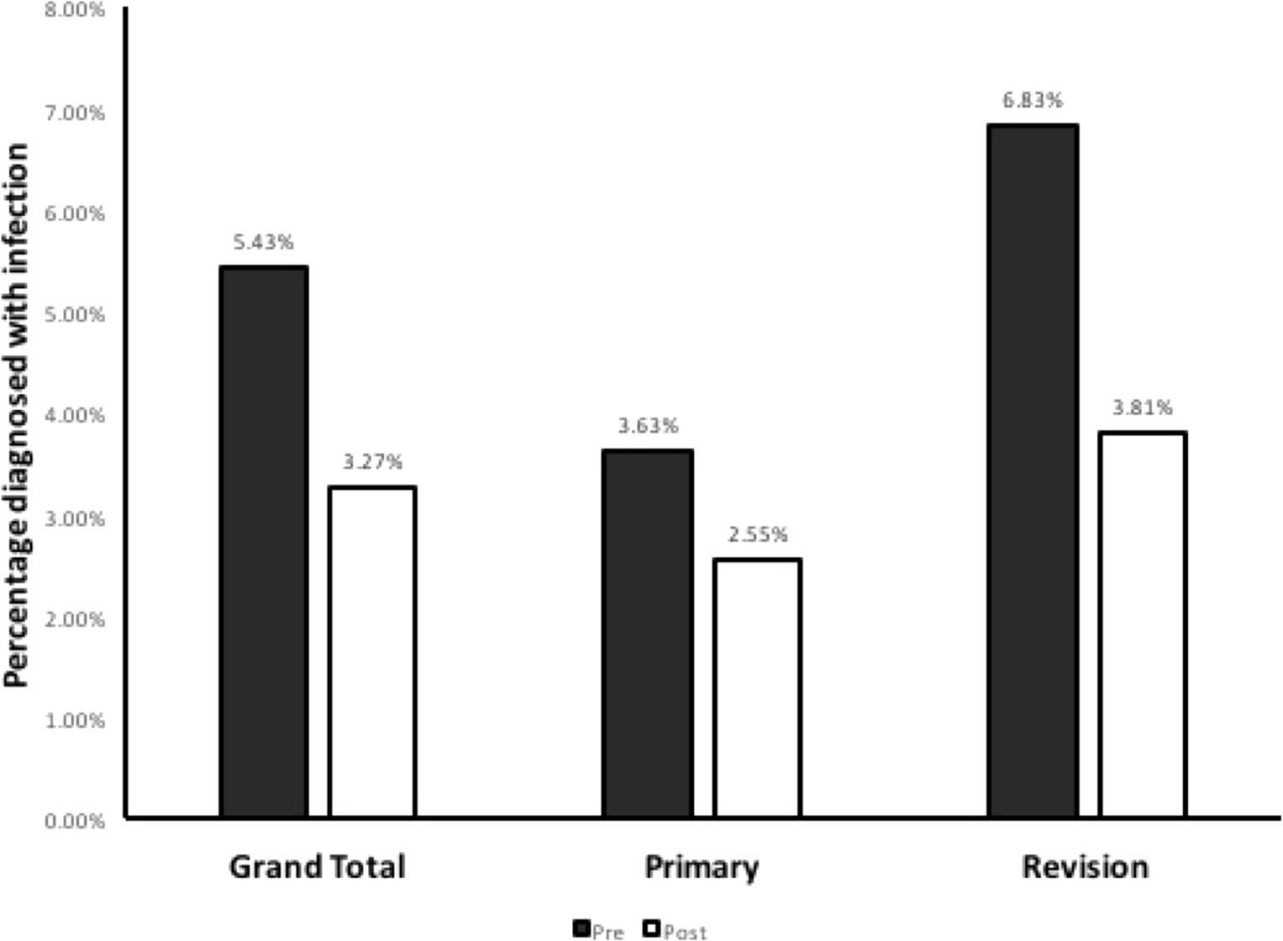
Overall CSF shunt infection rate: before (shaded bar) and after (open bar) introduction of protocol, in all cases (left), primary insertions only (centre), and revisional cases (right)

The overall infection rate decreased after the introduction of the protocol from 24 in 442 (5.43%) to 12 in 367 (3.27%), an absolute risk reduction (ARR) of 2.16%, with a relative risk reduction of 39.8% and the number needed to treat (NNT) to prevent one infection of 46.3 (Table 2, Fig. 6). However, chi-square analysis did not reach significance (*p* = 0.138).

**Table 2:** Infection rates before and after introduction of protocol, subdivided by type of procedure

Procedure type	Pre-protocol infection rate (%)	Post-protocol infection rate (%)	Relative risk	95% confidence interval
Total	24/442 (5.43%)	12/367 (3.27%)	0.602	0.31 to 1.19
Primary	7/193 (3.63%)	4/157 (2.55%)	0.702	0.21 to 2.36
Revision	17/249 (6.83%)	8/210 (3.81%)	0.558	0.25 to 1.27

For primary shunt insertions, the NNT was 92.6 (ARR = 1.08%, RRR = 29.8%), with chi-square analysis again not showing statistical significance (*p* = 0.565). The largest effect was observed in the shunt revision subgroup, where the NNT was 33.1 (ARR = 3.02%, RRR = 44.2%, *p* = 0.156).

### Regression analysis

No factor analysed using univariate binary regression against shunt infection showed an association (at the *P* ≤ 0.2 level) except for procedure subtype (primary or revision; *p* = 0.120), grade of operating surgeon (*p* = 0.016), and use of protocol (*p* = 0.142).

These were used in the multivariate model (Table 3) along with age at procedure. Age (*p* = 0.668) was included in the model despite not meeting this requirement due to the established association between low age and infection risk in literature. The surgeon grade was the only factor shown to be significantly associated with reduced risk of infection in this model (*p* = 0.028). Regression analysis showed an odds ratio of 0.581 in the post-protocol group compared to the pre-protocol group (*p* = 0.245), when adjusting for the other factors included in the analysis.

**Table 3:** Table demonstrating results of multivariate logistic regression. Odds ratios given are in comparison to baseline factor

Factor	Odds ratio (95% confidence interval)	Significance (*p* value)
Use of protocol	0.581 (0.285–1.184)	0.135
Surgeon grade (consultant vs. Res/Fell)	0.462 (0.232–0921)	0.028
Type of procedure		
Revision	1	
Primary	0.613 (0.283–1.330)	0.216
Age at procedure	0.999 (0.993–1.005)	0.745

### Compliance data

Compliance to the protocol was variable, with a range of 56% during the first month of introduction to 100% once it was established. Overall, 60% of the protocol checklists were returned. Of those, there was an average of 88% compliance. Compliance data was available for 10 of the 12 infection cases after the introduction of the protocol. There was only 1 documented protocol breach in a case that led to infection, which was incorrect prophylactic antibiotic administration in a patient in whom pre-operative screening skin and mucosa swabs had indicated methicillin-resistant *Staphylococcus aureus* (MRSA) carriage and antibiotic choice was not amended to account for this. Hence, there was no statistical association between protocol breaches and infection.

### Cultured organisms and latency

**Fig. 7:**
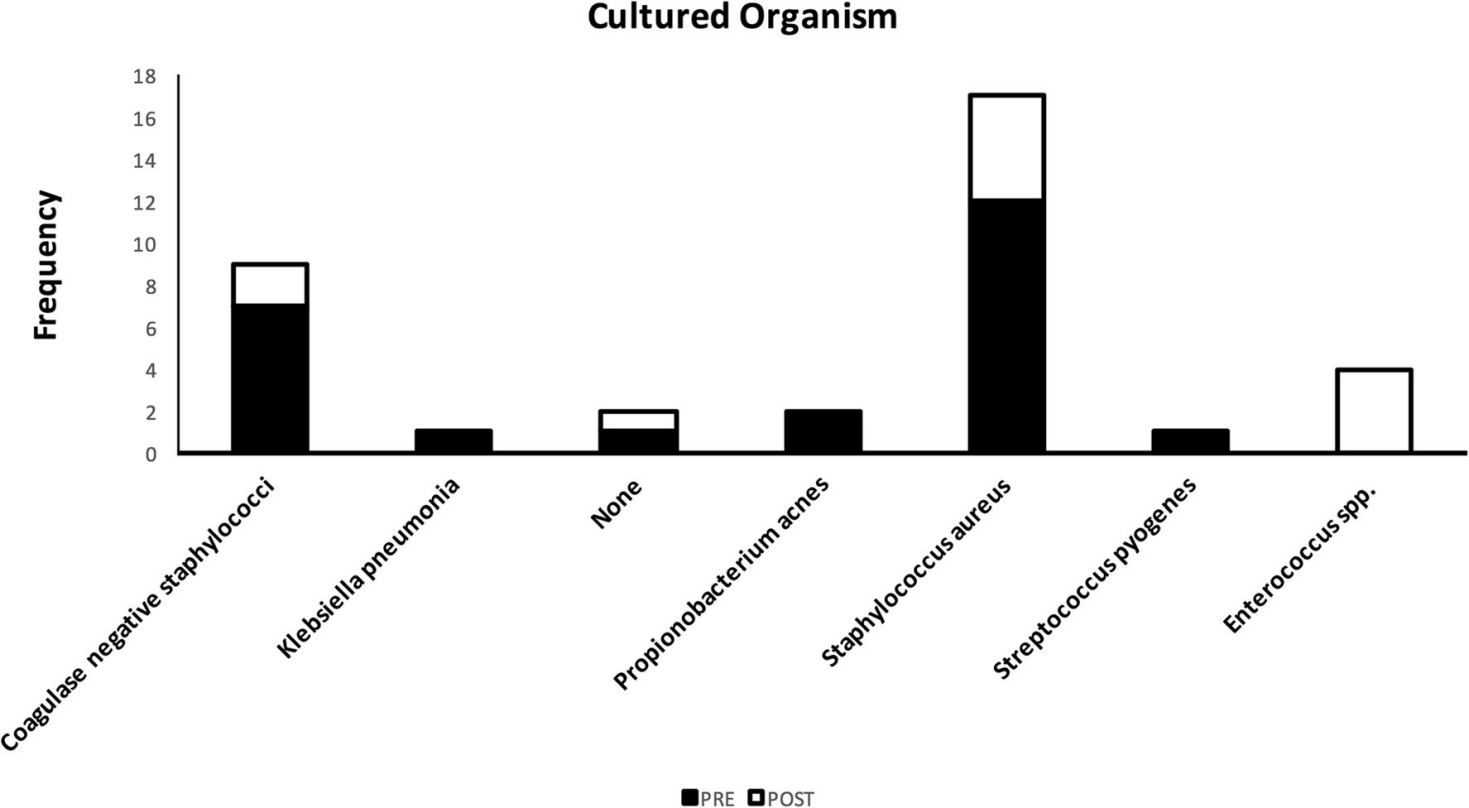
Causative pathogenic organisms in CSF shunt infections, before (shaded bar) and after (open bar) introduction on protocol

Of the 36 infections identified, 34 cultured a pathogenic organism with two patients treated empirically based on criteria discussed in the methods. CSF samples were the source of 27 of these cultured cases, with wound samples representing a further 7. The most commonly cultured organism was *Staphylococcus aureus* (Fig. 7, *n* = 17), followed by coagulase-negative staphylococci (*n* = 9).

This pattern was again seen in both the pre-protocol and post-protocol cohorts, although *Enterococcus faecalis* equalled coagulase-negative staphylococcus as the second most common cultured organism in the post-protocol group (*n* = 2).

In terms of latency, the average length of time between the date of the procedure and diagnosis of infection was 54.8 days. The pre-protocol group had an average latency of 67.3 days, compared to the post-protocol average of 29.9 days. However, two-tailed independent *t* test analysis did not demonstrate this difference to be statistically significant (*p* = 0.421).

## Discussion

The overall effect of the protocol in reducing the infection rate was similar to that seen in the literature [1, 7]. However, this reduction did not achieve statistical significance, which may be due to the contribution of a number of factors. Our institution’s infection rate was relatively low in the pre-protocol cohort to begin with, thereby requiring a large number of patients to achieve significance. In fact, the initial control infection rate demonstrated by the HCRN of 8.8% was reduced to 5.7% after the introduction of their protocol [8], which is 0.3% greater from our initial pre-protocol group’s 5.4% infection rate. Continuous review of the effects of the protocol as the number of procedures after the introduction of the protocol increases could yield significance.

There was a difference between the average ages between the pre- and post-protocol cohorts, which was not found to be statistically significant. Although this association was not demonstrated in our analysis at a statistically significant level (*p* = 0.745), young age is an established risk factor for infection [1, 13–15] and thus may have worked against the beneficial effect of the protocol. A relatively large difference was also noted between the proportion of patients in the post-protocol group with post-IVH as their presenting diagnosis compared to the pre-protocol group (18.0% compared to 28.6%), another factor described in literature as associated with infection [15], although this difference was not quite statistically significant (*p* = 0.06). Univariate regression analysis also did not show an association between infection and presenting diagnosis (*p* > 0.2).

Whilst the reduction did not reach statistical significance, the reduction in infection rate in both the primary and revision groups was encouraging—with NNTs of 92.6 (primary) and 33.1 (revisions)—suggesting that major units performing large numbers of such surgeries would see benefits from introducing similar protocols. With the large cost—both clinically and financially—of shunt infections, and a relatively simple and benign intervention of introducing a protocol, preventing 1 infection per 46.3 surgeries would appear to be a desirable effect.

Suboptimal compliance with the protocol may have had an effect on infection rate. A higher checklist return rate (60%) would allow investigation for associations between infection and individual components of the protocol. Care must be taken to improve checklist return and allow enhanced evaluation of the protocol.

Although the overall shunt infection rate is comparable, or even favourable, with the majority of large series in the literature, it is notable that it is still higher than the negligible rates achieved in two landmark series, from Paris [16] and Belgium [9]. In these series, all shunt surgeries were carried out on elective lists by a single senior surgeon. In the current series, operations were performed by a number of different surgeons at attending/consultant, fellow, and resident grades, with some cases being performed as ad hoc emergencies and some on planned elective lists. Surgeon seniority was a risk factor in the regression analysis of the current study, supporting the contention that the excellent rates achieved in the two cited studies depend on senior surgeon input. Differing health systems and clinical workloads internationally may affect the ability of individual institutions to achieve senior input for all shunt cases but we agree it is an ideal to strive towards.

Using this data, it may be possible to begin collaboration between different paediatric neurosurgical units to develop and implement a standardised protocol and establish a common baseline. This would benefit from a significantly larger sample size and hence allow more rapid analysis of the benefits, permitting adjustment where needed.

## Conclusion

Although the reduction in infection rates after the introduction of this protocol did not reach statistical significance, the reduction in overall infection rate in primary and revision shunt operations is highly promising, considering our initially low baseline infection rate. We recommend paediatric neurosurgical units consider introducing similar protocols for these procedures.

## References

[1] Rotim K, Miklic P, Paladino J, Melada A, Marcikic M, Scap M (1997). Reducing the incidence of infection in pediatric cerebrospinal fluid shunt operations. Childs Nerv Syst.

[2] Choksey MS, Malik IA (2004). Zero tolerance to shunt infections: can it be achieved?. J Neurol Neurosurg Psychiatry.

[3] Vinchon M, Dhellemmes P (2006). Cerebrospinal fluid shunt infection: risk factors and long-term follow-up. Childs Nerv Syst.

[4] Simon TD, Butler J, Whitlock KB, Browd SR, Holubkov R, Kestle JRW, Kulkarni AV, Langley M, Limbrick DD, Mayer-Hamblett N, Tamber M, Wellons JC, Whitehead WE, Riva-Cambrin J (2014). Risk factors for first cerebrospinal fluid shunt infection: findings from a multi-center prospective cohort study. J Pediatr.

[5] Vinchon M, Rekate H, Kulkarni AV (2012). Pediatric hydrocephalus outcomes: a review. Fluids Barriers CNS.

[6] Tamber MS, Klimo P, Mazzola CA, Flannery AM (2014). Pediatric hydrocephalus: systematic literature review and evidence-based guidelines. Ref 8: Management of cerebrospinal fluid shunt infection. J Neurosurg Pediatr.

[7] Kestle JRW, Riva-Cambrin J, Wellons JC, Kulkarni AV, Whitehead WE, Walker ML, Jerry Oakes W, Drake JM, Luerssen TG, Simon TD, Holubkov R (2011). A standardized protocol to reduce cerebrospinal fluid shunt infection: the Hydrocephalus Clinical Research Network Quality Improvement Initiative. J Neurosurg Pediatr.

[8] Kestle JRW, Holubkov R, Cochrane DD, Kulkarni AV, DDL J, Luerssen TG, Oakes WJ, Riva-Cambrin J, Rozzelle C, Simon TD, Walker ML, III JCW, Browd SR, Drake JM, Shannon CN, Tamber MS, Whitehead WE, Network THCR (2016). A new Hydrocephalus Clinical Research Network protocol to reduce cerebrospinal fluid shunt infection. J Neurosurg Pediatr.

[9] Pirotte BJM, Lubansu A, Bruneau M, Loqa C, Van Cutsem N, Brotchi J (2007). Sterile surgical technique for shunt placement reduces the shunt infection rate in children: preliminary analysis of a prospective protocol in 115 consecutive procedures. Childs Nerv Syst.

[10] Ratilal B, Costa J, Sampaio C (2006) Antibiotic prophylaxis for surgical introduction of intracranial ventricular shunts. Cochrane Database Syst Rev CD005365. 10.1002/14651858.CD005365.pub2 10.1002/14651858.CD005365.pub2 PMC8407112 16856095

[11] Rehman AU, Rehman TU, Bashir HH, Gupta V (2010). A simple method to reduce infection of ventriculoperitoneal shunts. J Neurosurg Pediatr.

[12] Webster J, Osborne S (2007) Preoperative bathing or showering with skin antiseptics to prevent surgical site infection. Cochrane Database Syst Rev CD004985 10.1002/14651858.CD004985.pub4 22972080

[13] Ammirati M, Raimondi AJ (1987). Cerebrospinal fluid shunt infections in children. A study on the relationship between the etiology of hydrocephalus, age at the time of shunt placement, and infection rate. Childs Nerv Syst.

[14] George R, Leibrock L, Epstein M (1979). Long-term analysis of cerebrospinal fluid shunt infections. A 25-year experience. J Neurosurg.

[15] Dallacasa P, Dappozzo A, Galassi E, Sandri F, Cocchi G, Masi M (1995). Cerebrospinal fluid shunt infections in infants. Childs Nerv Syst.

[16] Choux M, Genitori L, Lang D, Lena G (1992). Shunt implantation: reducing the incidence of shunt infection. J Neurosurg.

